# Bridging Hierarchies in Multi-Scale Models of Neural Systems: Look-Up Tables Enable Computationally Efficient Simulations of Non-linear Synaptic Dynamics

**DOI:** 10.3389/fncom.2021.733155

**Published:** 2021-10-01

**Authors:** Duy-Tan J. Pham, Gene J. Yu, Jean-Marie C. Bouteiller, Theodore W. Berger

**Affiliations:** Department of Biomedical Engineering, Center for Neural Engineering, University of Southern California, Los Angeles, CA, United States

**Keywords:** input-output modeling, multi-scale modeling, large-scale modeling, glutamatergic receptors, look-up table, synapse, AMPAR, NMDAR

## Abstract

Synapses are critical actors of neuronal transmission as they form the basis of chemical communication between neurons. Accurate computational models of synaptic dynamics may prove important in elucidating emergent properties across hierarchical scales. Yet, in large-scale neuronal network simulations, synapses are often modeled as highly simplified linear exponential functions due to their small computational footprint. However, these models cannot capture the complex non-linear dynamics that biological synapses exhibit and thus, are insufficient in representing synaptic behavior accurately. Existing detailed mechanistic synapse models can replicate these non-linear dynamics by modeling the underlying kinetics of biological synapses, but their high complexity prevents them from being a suitable option in large-scale models due to long simulation times. This motivates the development of more parsimonious models that can capture the complex non-linear dynamics of synapses accurately while maintaining a minimal computational cost. We propose a look-up table approach that stores precomputed values thereby circumventing most computations at runtime and enabling extremely fast simulations for glutamatergic receptors AMPAr and NMDAr. Our results demonstrate that this methodology is capable of replicating the dynamics of biological synapses as accurately as the mechanistic synapse models while offering up to a **56-fold increase in speed**. This powerful approach allows for multi-scale neuronal networks to be simulated at large scales, enabling the investigation of how low-level synaptic activity may lead to changes in high-level phenomena, such as memory and learning.

## Introduction

In modern neuroscience, computational modeling has become a pivotal part of research as it allows for the investigation of underlying physiological neural mechanisms that are often too difficult to test experimentally on live tissue. Multi-scale and large-scale models of the nervous system aim to replicate and integrate complex dynamics across multiple hierarchies (e.g., molecular, synaptic, single neuron, neuronal network) within large networks of neurons. Such models have the potential to further our understanding of how low-level mechanisms (such as biomolecular interactions) might affect high-level outcomes, such as cognition (Micheli et al., 2021). Depending on the level of analysis being performed, large-scale models can integrate over multiple temporal and spatial scales, with varying degrees of detail as to keep simulations feasible, tractable, and adequately informing. On the less detailed end of the spectrum, one can avoid the heavy complexity of simulating many individual neurons by condensing them into neural ensembles that are reduced to statistical descriptions as done in the use of the Fokker-Planck equation (FPE)—which imposes the “diffusion approximation” simplification—or through neural mass models (NMMs) that describe the mean activity of neural populations as described in Breakspear (2017). In efforts to bridge micro- to macroscopic scales, others have employed simple neural activation functions to model neuromodulation across the brain as described in Shine et al. (2018, 2021) or by stacking each hierarchical scale with feedforward *ad hoc* models, such that each scale is modeled individually, to examine how changes in molecular parameters impact neuronal firing rates (Bouteiller et al., 2011). Multi- and large-scale models of entire neural subsystems that are anatomically detailed with connectivity or that consider cell morphology closely tend to require entire clusters of computing nodes to simulate (Izhikevich and Edelman, 2008; Yu et al., 2013). These multi-scale models have potential for *in silico* experimentation of neurological perturbations either due to specific pathologies, electrical stimulation (electrotherapy), or the influence of exogenous compounds (i.e., drugs), thereby constituting a useful platform for the discovery and development of novel therapeutics. This has important implications in neuropharmacology, for example, as it enables parts of the drug discovery pipeline to be performed via simulations, potentially resulting in a massive speedup in the drug development process. Yet, for multi- and large-scale models to attain such predictive power, they must provide a sufficiently accurate representation of the underlying neuronal processes, making it necessary to depict their constituents, at all levels, with biological accuracy.

Various methods in accelerating simulations have been explored to alleviate the long runtimes experienced in large-scale neuronal models. In Marasco et al. (2012), entire complex cell morphologies were reduced to few functionally equivalent compartments that can accurately reproduce the electrophysiological properties of full morphological cell models with 13–50 times faster runtimes. Tikidji-Hamburyan and Colonnese (2021) used combinations of polynomial, piecewise-linear, and step (PLS) functions to accurately approximate neuronal dynamics resulting in speedups ranging from 3 to 5 times faster than the original models. Multiple *artificial* neural network (ANN) architectures were explored in Olah et al. (2021) where runtimes in networks containing 50 neuronal cells were accelerated by orders of magnitude compared to their traditional modeling counterparts.

The high-level outcomes of a neural system model that spans multiple hierarchies is highly contingent on the processes of its lower-level components. That is, the observed macroscale behavior of such a model will depend on the microscale activity whose effects will propagate up the hierarchical scales. One of the fundamental levels in hierarchically modeling neural systems arguably lies at the synaptic scale. Not only do synapses form the basis for neuronal transmission, they are also critical in modulating learning and memory (Bliss and Collingridge, 1993; Mayford et al., 2012). Because of the highly impactful role that synapses play, their accurate representation is critical.

Several studies have shown the success of mechanistic approaches that use Markov kinetic state models (Robert and Howe, 2003; Schorge et al., 2005). Such kinetic models represent synaptic dynamics using multiple internal states that are governed by multiple ordinary differential equations (ODEs) and can capture the complex non-linear and time-varying behavior of synaptic mechanisms. However, in the context of large-scale modeling, these methodologies become increasingly prohibitive due to their computational burden. As a result, large network simulations involving kinetic models of synapses lead to extremely long runtimes given the sheer number of synapses that must be simulated. To compensate, synaptic dynamics are often reduced to linear rise and decay exponential functions (alpha synapses) due to their small computational load. These alpha synapses alleviate the computational complexity problems of kinetic models by providing fast simulation times, but they are unable to capture any level of non-linearity and thus, are inadequate at accurately replicating complex synaptic dynamics observed experimentally.

Previously, our group had proposed the Laguerre-Volterra input-output (LVIO) model (Hu et al., 2015) and its artificial neural-network variant, the Laguerre-Volterra Network (LVN) (Hu et al., 2018) as faster, middle-ground alternatives to synaptic modeling. These two models aim to efficiently map the mathematical relationship between inputs and outputs of synapses while avoiding the modeling of the underlying complex biological mechanisms. Both models involve combining the Volterra functional power series with Laguerre basis functions (Marmarelis, 1993), thereby replacing the complex computations associated with solving multiple ODEs in the kinetic models with simple computations involving algebraic equations (for details, see Hu et al., 2015, 2018). Despite its speed advantage over kinetic models, the latter LVN model still runs notably slower than the alpha synapse model, which provides room for even further improvement. Here, we introduce a novel look-up table synapse (LUTsyn) model for both ionotropic receptors, AMPAr and NMDAr, of glutamatergic synapses that further reduces simulation time while replicating kinetic model dynamics. Similar to its Laguerre-Volterra predecessors, the LUTsyn model implements the input-output relationship of synaptic dynamics by way of look-up tables without simulating the computationally heavy mechanisms.

In this paper, we first introduce three underlying assumptions imposed by the LUTsyn model, illustrate the structure of the model, describe how it operates at runtime, and discuss how different structural parameters may affect its performance. We then cover how mechanistic kinetic synapse models (for AMPAr and NMDAr) serve as ground truth input-output data and how they are utilized to populate the look-up table with amplitude values. The look-up table data structure is further discussed, addressing issues of indexing and size followed by a description of the implementation of the models into the NEURON simulation environment. Then, the methodology for optimizing the time constants of the model is covered as well as the equations involved during runtime. Using the kinetic models as reference, we then validate (1) the LUTsyn model’s accuracy in response to random pulse train inputs and (2) its ability to reproduce precise spike times by measuring spike synchrony at both the single cell level and network level. To measure the speed of the model, we implement a large-scale neuronal network simulation—where each receptor uses the LUTsyn model—and record the runtime. We compare these benchmarks to other synapse models (kinetic, LVN, and exponential) and show the significant speed increase that our new model delivers while offering powerful predictability on a complex non-linear system. Our results strongly demonstrate that our LUTsyn methodology enables large-scale network-level simulations containing a very large number of synapses, all while maintaining biological realism at high speeds.

## Materials and Methods

The LUTsyn model is a methodology that aims to abstract the input-output relationships for the glutamatergic receptors AMPAr and NMDAr in a computationally inexpensive way. The implementation of the LUTsyn model was achieved by imposing three major simplifying assumptions:

1. The input to the model—presynaptic neurotransmitter release or “pulse”—is a binary time series.
2. The temporal waveform underlying any *N*th order output response is fixed. That is, the normalized response to any input is identical to other normalized responses of the same order.
3. The amplitude of the output waveform is modulated based on the relative timings of previous inputs (interpulse intervals) within a finite memory window.

Assumption 1 can safely be made due to the quantal nature of neurotransmitter release (del Castillo and Katz, 1954). Because neurotransmitter is released in discrete vesicles (i.e., fixed amount) in response to presynaptic cells firing, only the timings of presynaptic events need to be accounted for, not the amount of neurotransmitter. Assumption 2 enables our model to greatly simplify the output by providing a computationally light template waveform in response to each presynaptic input event. Since AMPAr and NMDAr can accurately be characterized by kinetic state models (Robert and Howe, 2003; Schorge et al., 2005), assumption 3 can be made without major loss in accuracy because of the behavior of these kinetic models: past input events (i.e., “pulses”) that occur closer in time to the present input event have a larger effect on the amplitude of the present output, whereas input events that occur further in the past have a diminishing effect. Therefore, the amplitude of the present output waveform is assumed to be a function of the temporal pattern of past input events—i.e., the past interpulse intervals. Moreover, the LUTsyn model assumes a finite memory window where input events that occur far enough in the past will have no effect on the present output. With these assumptions, the LUTsyn model stores precomputed amplitude values into an array-like data structure (i.e., the look-up table) such that access to these values is accomplished by using the interpulse intervals of past input events as indices.

### LUTsyn Model Structure

**FIGURE 1:**
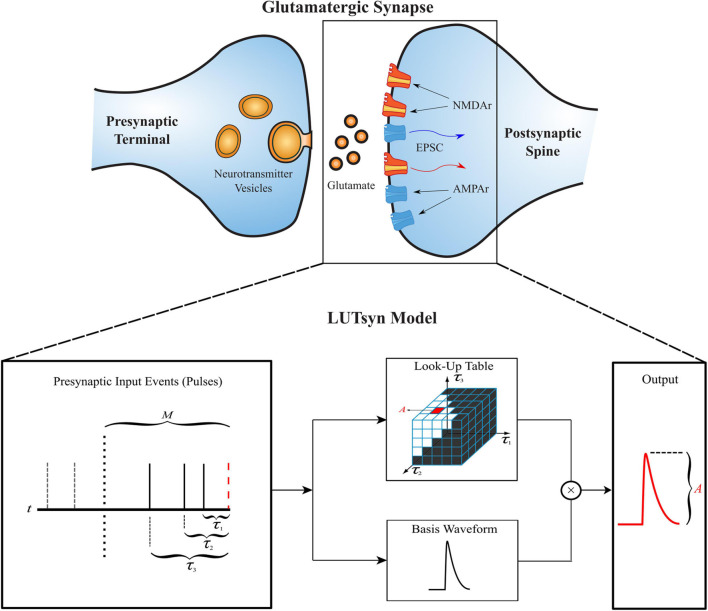
Structure of the LUTsyn model. The LUTsyn model is an input-output model of glutamatergic synapses containing AMPAr and NMDAr. The input to the model is a binary time series representing the times when neurotransmitter is released by a presynaptic terminal. The model records past input events that occur within a specified memory window (denoted by *M* and shown as the dotted vertical line) and calculates a finite number of interpulse intervals (denoted by *τ* and shown as the solid vertical lines) to predict the output response of the present input event (red dashed line). All input events beyond the memory window (shown as the two dashed vertical lines) are ignored in the prediction of the present output. The interpulse intervals are used to access a look-up table which contains precomputed amplitude values. The value retrieved from the look-up table is multiplied by a normalized basis waveform to compute the prediction of the output response to the present input pulse.

The LUTsyn model’s structure is illustrated in Figure 1. The model takes a binary time series as input, indicating presynaptic neurotransmitter release, and provides a continuous time series as output, representing either AMPAr conductance or NMDAr open-state probability. The three simplifying assumptions described above lead to two fundamental objects in the LUTsyn model. The first (based on assumption 2) is the *basis waveform* which serves as a template, providing the shape to each output. It is derived from an exponential synapse model whose output is optimized via time constants to estimate the output response of a kinetically modeled synapse and normalized to have an amplitude of 1 (details are discussed in the Optimization of Time Constants section). The second fundamental object is the *look-up table* (note: we will use “look-up table” to refer to the data structure and “LUTsyn” to refer to the model) in which the amplitude values are stored in a multi-dimensional array-like data structure that is indexed by past input interpulse intervals (IPIs). The look-up table provides the predicted amplitude of a given output waveform such that the amplitude value is multiplied with the basis waveform to provide the model’s output response. At runtime, the LUTsyn model operates by progressing through the simulation and searching for presynaptic input events. When an input event is received, the model will calculate the IPIs of past input events which will be used as indices for the look-up table to retrieve the corresponding amplitude value for the present pulse’s output response. The present pulse time is then stored to be used in future IPI calculations. The past IPIs are denoted as *τ* (*τ*_1_, *τ*_2_, etc.). For example, *τ*_1_ would represent the IPI between the present pulse and the most recent past pulse, and *τ*_2_ would represent the IPI between the present pulse and the second most recent past pulse. These *τ* values are transformed into a single index that accesses the corresponding look-up table amplitude value (see Look-Up Table Size and Indexing section).

The LUTsyn model contains multiple parameters that affect both its memory footprint and performance. The **order** of the model dictates the number of past pulses that are considered to form the predicted amplitude value. For example, a LUTsyn model of order 4 considers four pulses: the present pulse and the three most recent pulses. In other words, a 4th order LUTsyn model is indexed using *τ*_1_, *τ*_2_, and *τ*_3_ (3 IPIs). As a result, the order will also determine the dimension of the look-up table (*N*th order implies an *N*-1 dimensional look-up table) which directly governs the memory size. As the order of the look-up table increases, the accuracy of the model will also increase because more pulse interactions are accounted for. However, because input pulses that occur further in the past have decreasing effects on the present output, the marginal improvement of the model’s accuracy will decrease as the model’s order increases. Therefore, the order must be chosen to balance model accuracy and memory consumption. Note: this paper refers to two different, yet related, notions of “order.” The first is used as defined in this paragraph—the maximal number of past pulses considered for a particular LUTsyn model. The second is a way to describe responses that occur as a result of successive input pulses (e.g., a second order response is one that resulted from two successive input pulses).

The **memory window**, *M* (measured in milliseconds), is another parameter which determines how far in the past the LUTsyn model will look for input events. Pulses that occur beyond the memory window are assumed to have no effect on the present pulse’s output response and are therefore ignored. Consequently, when an amount of time greater than *M* has passed without any input events, the next input event will be treated as a “first order pulse.” Additionally, the memory window governs the size of the look-up table because it is the upper limit of *τ*. Increasing *M* may improve accuracy by considering more temporally distant pulse interactions but will also increase memory size. Again, the value of *M* must be chosen to balance accuracy and memory consumption.

The **granularity**, denoted by *δ*, determines the resolution of IPIs for which the look-up table is constructed. For example, a 2nd order look-up table (indexed with just *τ*_1_) with a memory window size of 500 ms and a granularity of 5 ms will contain 100 values, for which each subsequent value is sampled in increments of 5 ms. The IPI values are rounded to the closest multiple of *δ* when generating the prediction index. For the previous example, if *τ*_1_ = 92 ms, then the index that corresponds to a 90 ms delay (closest multiple of 5 ms) is used. The value of *δ* should be chosen such that *δ* is a divisor of the memory window *M*. Decreasing the value of *δ* results in finer resolution which may increase accuracy but will also increase the look-up table’s memory consumption due to more values being stored.

The use of exponential functions as the template of the output requires the optimization of **time constants** (*T*_*c*_), which govern the temporal dynamics and shape of the model’s predicted waveform. These time constants must be optimized such that the dynamics of the kinetic synapse models can be replicated. Unlike the other parameters, the choice of time constants has no trade-off between model accuracy and memory consumption.

### LUTsyn Model Equations

The LUTsyn model utilizes a triple exponential function (sum of three exponentials) to serve as the basis of the temporal output waveform to replicate the kinetic synapse models. The triple exponential function was used to model the output response to a single input event, which was parameterized by three time constants *T*_*c1*_, *T*_*c2*_, and *T*_*c3*_ (one for each exponential) shown in the following equation:


(1) $$\hat{y}\,\left(t\right)=F_{n\,o\,r\,m}\cdot \left(w\cdot e^{\frac{-t}{T_{c\,2}}}+\left(1-w\right)\cdot e^{\frac{-t}{T_{c\,3}}}-e^{\frac{-t}{T_{c\,1}}}\right)$$

for which $\hat{y}\,\left(t\right)$ represents the general normalized output response of the LUTsyn model, which is either a measure of conductance (for AMPAr) or a measure of open-state probability (NMDAr). *F*_*norm*_ is a normalization factor that scales the amplitude of the waveform to be 1 (based on the triplet of time constants used), *t* represents the amount of time that has passed since the input event (in ms), and *T*_*c1*_, *T*_*c2*_, and *T*_*c3*_ are the time constants (ms) that govern the dynamics of the waveform which are optimized to replicate the output waveform of the kinetic models. The *w* variable is a weighting factor constrained within the range (0,1) and was optimized (along with the time constant variables via grid search) to have a value of ∼0.963 for both AMPAr and NMDAr. *F*_*norm*_ values were computed empirically once the time constants were known.

Ultimately, the LUTsyn models must output conductance values for each receptor to simulate the receptor-mediated excitatory postsynaptic currents (EPSCs). The following equations show the calculations involved in the LUTsyn model at runtime:


(2) $$I_{r}\,\left(t\right)=n\,b_{r}\cdot \left(V-V_{r\,e\,v}\right)\cdot g_{r}\,\left(t\right)$$

(3) $$g_{A\,M\,P\,A}\,\left(t\right)=L\,U\,T_{A\,M\,P\,A}\times {\hat{y}}_{A\,M\,P\,A}\,\left(t\right)$$

(4) $$O_{N\,M\,D\,A}\,\left(t\right)=L\,U\,T_{N\,M\,D\,A}\times {\hat{y}}_{N\,M\,D\,A}\,\left(t\right)$$

(5) $$g_{0}=g_{1}\,\frac{g_{2}-g_{1}}{1+e^{\alpha \,\psi_{m}}}$$

(6) $$g_{m\,a\,x}=\frac{g_{0}}{1+\left(\frac{M\,g_{0}^{2+}}{K_{0}}\right)\,e^{-0.062\cdot V}}$$

(7) $$g_{N\,M\,D\,A}\,\left(t\right)=g_{m\,a\,x}\times O_{N\,M\,D\,A}\,\left(t\right)$$

for which *I_r*, *nb_r*, and *g_r* represent EPSC, number, and conductance of receptor *r* (AMPAr or NMDAr), respectively. *V* represents the membrane voltage, and *V*_*rev*_ represents the reversal potential (set to 0 mV for both receptors). *g*_*AMPA*_ and *g*_*NMDA*_ are the single-receptor conductances of AMPAr and NMDAr, respectively. *LUT*_*AMPA*_ and *LUT*_*NMDA*_ are the amplitude values (for each respective receptor) that scale—depending on the present IPI pattern—the normalized triple exponential waveforms of ${\hat{y}}_{A\,M\,P\,A}$ and ${\hat{y}}_{N\,M\,D\,A}$, which both take the form of Equation **1**. *O*_*NMDA*_ represents the open-state probability of NMDAr. Equation **5** was derived from Ambert (2010) for which *g_0* represents the total NMDAr conductance in the absence of magnesium and α represents the steepness of the voltage-dependent transition from *g_1* to *g_2*. *g_1* and *g_2* represent the open state conductances with one and two glutamate molecules bound with values of 40 and 247 pS, respectively. Equation **6** was derived from Jahr and Stevens (1990) for which $M\,g_{0}^{2}$ represents the external magnesium concentration (with value of 1 mM) and *K_0* represents the equilibrium constant of magnesium (set to 3.57).

### Kinetic Synapse Models for Glutamatergic Receptors AMPAr and NMDAr

Our proposed LUTsyn approach is an IO model that fundamentally relies on data that accurately represents the system being modeled. Despite their computational burden, kinetic models provide value in their realistic portrayal of biological systems and thus, were used as the source of input-output data for which the LUTsyn model was based. Specifically, two different kinetic synapse models were utilized—one for each of the ionotropic receptors, AMPAr and NMDAr—to generate the look-up tables. The AMPAr model, derived from Robert and Howe (2003), consists of 16 internal states and outputs the conductance of the AMPAr-associated channel. The NMDAr model, derived from Schorge et al. (2005), consists of 15 states and uses the receptor’s open-state probability as output (see Supplementary Figures 1, 2 for kinetic state model schematics). Both kinetic models use glutamate concentration (in response to a presynaptic cell firing) as input, which is calculated using the NTDiffusion model based on the analytic solutions for neurotransmitter diffusion in the cleft by Savtchenko and Rusakov (2007). The NTDiffusion model uses nominal parameter values of a 60 nm cleft radius, a 20 nm cleft height, and a diffusivity of 0.33 μm^2^ ms^–1^. These models were tuned to represent the synaptic connections from the entorhinal cortex to the dentate gyrus in rat hippocampus. For AMPAr, parameters were chosen that nominally induce a 0.22 mV excitatory postsynaptic potential (EPSP) at the soma in accordance with Foster et al. (1991). The rate constants for both AMPAr and NMDAr kinetic models are summarized in Supplementary Tables 1, 2, which are taken (and modified for NMDAr) from Allam et al. (2012).

### Look-Up Table Generation

**FIGURE 2:**
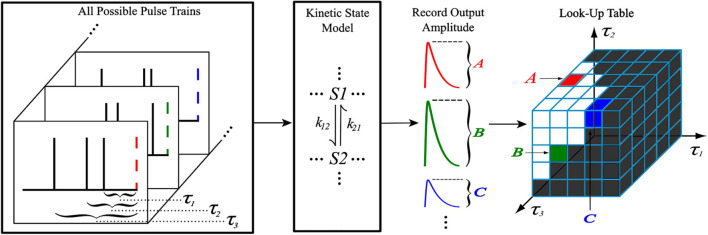
Generation of a 4th order look-up table. For a 4th order look-up table, all possible temporal combinations of input pulse trains consisting of four pulses (constrained by *M* and *δ*) are sent to a kinetic state model (either representing AMPAr or NMDAr). For each input pulse train, the amplitude of the kinetic model’s output response due to the fourth pulse is recorded and stored into the look-up table at an index that corresponds to the IPIs of the pulse train.

The generation of the look-up tables utilized the kinetic models discussed previously with an iterative process shown in Figure 2. When generating an *N*th order look-up table, a train of *N* pulses (with IPIs *τ*_1_, *τ*_2_, …, *τ_*N*_*_–__1_) was sent as input to the kinetic model where the amplitude of the *N*th pulse’s output response was recorded and stored. This process was repeated with a new input train until every combination of *τ*_1_, …, *τ_*N*_*_–__1_ was exhausted, which was constrained by the memory window and granularity parameters. Each amplitude value of the look-up table was stored at an index that corresponds to the *τ* values that were used to obtain it, making access simple. Because the time courses of AMPAr and NMDAr are inherently different, the LUTsyn model parameters were chosen specifically for each receptor. AMPAr has faster dynamics than NMDAr which results in a finer granularity but smaller memory window. We chose the look-up table to be of 4th order with a memory window of 300 ms and a granularity of 1 ms for AMPAr. The 1 ms granularity provided sufficient accuracy to the model while keeping memory consumption low. See Supplementary Figure 3 for a comparison on the effects on accuracy and memory of different granularity levels. NMDAr exhibits much slower dynamics, greater non-linearity, and longer lasting pulse interactions. We set the NMDAr look-up table to be of 5th order with a memory window of 1,000 ms and a granularity of 5 ms. Both memory window values were found by searching for the amount of time required between two pulses (simulated on the kinetic models) such that the second pulse’s output response had an amplitude within a 2% error of the first, which would signify that the system had returned to a “baseline” state after transients from the first pulse settled down.

### Look-Up Table Size and Indexing

**FIGURE 3:**
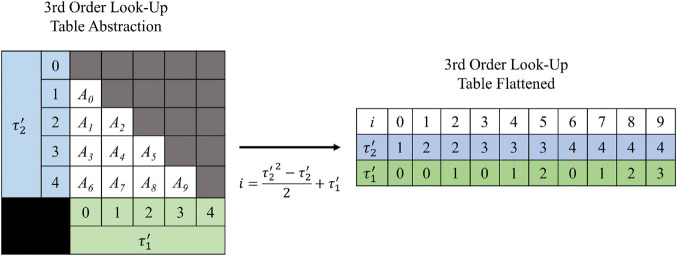
Visualization of 3rd order look-up table. A 3rd order look-up table can be abstracted as a two-dimensional data structure indexed by **$\tau_{1}^{{}^{\prime }}$** and **$\tau_{2}^{{}^{\prime }}$** (integer analogs of the IPIs **_*τ***1**_** and **_*τ***2**_**). The intrinsic constraint **_*τ***1**_** < **_*τ***2**_** for a given pulse train gives the look-up table its “lower triangular matrix” shape (shown on the left) where *A*_*i*_ represents the *i*th amplitude value stored. For efficient use of computer memory, the look-up table is flattened into a one-dimensional array (shown on the right) which is indexed by *i*. The one-dimensional index *i* has a direct mathematical mapping from the two-dimensional indices **$\tau_{1}^{{}^{\prime }}$** and **$\tau_{2}^{{}^{\prime }}$** (shown by the center arrow) which was derived by Equation **10** and taking *N* = 3.

The shape of the look-up tables are not uniform hypercubes (square, cube, tesseract, etc.) for which each *τ* axis contains the same number of values. This is because of the intrinsic constraint *τ*_1_ < *τ*_2_ < … < *τ_*N*_*_–__1_. Figure 3 illustrates the shape of the look-up table data structure for a 3rd order (2-dimensional) LUTsyn model. As a result of the shape, the look-up table was stored as a sequentially flattened one-dimensional array such that an index transformation is used to access the values. The following Equations **8, 9** are the general form for calculating the number of values in an *N*th order look-up table with memory window *M* and granularity *δ*:


(8) C⁢(R,N)=(RN-1)=R!(N-1)!⁢(R-N+1)!

(9) $$R=\frac{M}{\delta },R\in \mathbb{N}$$

for which *R* represents the ratio (assumed to be a positive integer) between the memory window (*M*) and granularity (*δ*). *R* can be interpreted as the maximum number of time samples within a memory window given a specific granularity. *C(R,N)* represents the total number of values stored in a look-up table of *N*th order, with ratio *R*. Equation **8** is equal to the *combination* function. It can be interpreted as having *R* time slots and any *N*-1 of those slots contains an input event: “*R* choose *N*-1.” The look-up tables reported in this paper are of 4th (for AMPAr) and 5th (for NMDAr) order. The specific formulas for calculating the number of values in 4th and 5th order look-up tables, as functions of *R*, were derived by plugging in *N* = 4 and *N* = 5 into Equation **8** and simplifying:


(8.1) $$C\,\left(R,4\right)=\frac{R^{3}-3\,R^{2}+2\,R}{6}$$

(8.2) $$C\,\left(R,5\right)=\frac{R^{4}-6\,R^{3}+11\,R^{2}-6\,R}{24}$$

for which *C(R,4)* and *C(R,5)* represent the total number of values stored in 4th and 5th order look-up tables, respectively. The *R* value for AMPAr is 300 (*M* = 300 ms, *δ* = 1 ms) while the *R* value for NMDAr is 200 (*M* = 1,000 ms, *δ* = 5 ms). Using the equations above, the total number of values in the 4th order AMPAr look-up table and the 5th order NMDAr look-up table are 4,455,100 and 64,684,950, respectively. Since each value stored is of datatype double, then the memory size, in bytes, can be calculated by multiplying the number of values, *C*, by 8. This results in the 4th order AMPAr look-up table consuming 35,640,800 bytes (∼33.9 MB) of memory while the 5th order NMDAr look-up table consumes 517,479,600 bytes (∼493.5 MB). Generation of the look-up tables was parallelized and performed on a high performance computer cluster containing 1,000 processing cores (Intel Xeon 4116). The time required to generate the AMPAr and NMDAr look-up tables were ∼24 and ∼299 min, respectively.

The general equation that converts the multi-dimensional indices (using *τ* values) into a one-dimensional index were derived using Equation **8**:


(10) $$i_{N}\,\left(\tau_{1}^{\prime },\tau_{2}^{\prime },\ldots ,\tau_{N-1}^{\prime }\right)=\sum_{n=1}^{N-1}C\,\left(\tau_{n}^{\prime },n\right)$$

(11) $$\tau_{n}^{\prime }\,\left(\tau_{n},\delta \right)=r\,o\,u\,n\,d\,\left(\frac{\tau_{n}}{\delta }\right)$$

for which *δ* represents the granularity of the LUTsyn model (measured in ms), the *round*() function rounds the input to the nearest integer, *τ*_*n*_ represents the interpulse interval between the *n*th most recent input pulse and the present input pulse in milliseconds, $\tau_{n}^{\prime }$ represents the transformation of *τ*_*n*_ into an array index (which must be a natural number) that depends on the value of *δ*, and *i*_*N*_ is the *N*th order one-dimensional index transformation given the multi-dimensional indices τ,′1τ,′2…,τN-1′. For 4th and 5th order look-up tables, the formulas for the one-dimensional indices were derived by plugging in *N* = 4 and *N* = 5 into Equation **10**:


(10.1) i4(τ,′1τ,′2τ)′3=τ3 3′-3⁢τ3 2′+2⁢τ3′6+τ2 2′-τ2′2+τ1′

(10.2) i5(τ,′1τ,′2τ,′3τ)′4=τ4 4′-6⁢τ4 3′+11⁢τ4 2′-6⁢τ4′24+i4(τ,′1τ,′2τ)′3

### Implementation in NEURON Framework

The LUTsyn, kinetic, and neurotransmitter diffusion models were all implemented into NEURON, a simulation environment for computational models of neuronal networks (Hines and Carnevale, 1997). Each model was written into a module (.mod) file using the NMODL programming language. In this study, large-scale simulations, model validation, and look-up table generation were all performed using the NEURON framework (v7.6.7). NEURON simulations were interfaced through the Python programming language (v3.5) and allowed for multiple instantiations of each model to run in parallel. The LUTsyn models of AMPAr and NMDAr were implemented in separate .mod files and were written to follow the logic described in the previous LUTsyn Model Structure section. In order for NEURON to interface with the data structures of the look-up tables, the look-up tables are first loaded in Python as NumPy arrays. Access to these arrays was achieved by creating pointers that held their memory addresses and linking them to every NEURON instantiation of the LUTsyn model. In the context of large-scale simulations, this is a highly memory efficient approach as only one look-up table must be loaded into memory (per core) for any number of instantiations of the synapse to access them.

### Optimization of Time Constants

**FIGURE 4:**
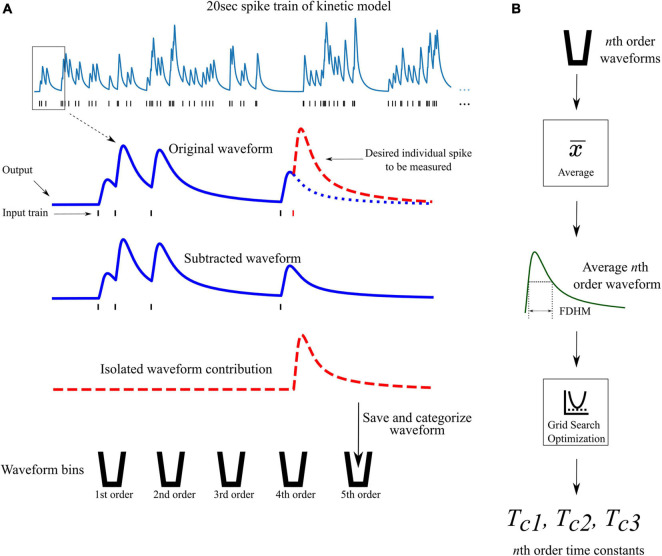
Waveform isolation and time constant optimization. **(A)** A 20 s 10 Hz Poisson random pulse train response (simulated using the kinetic models) is decomposed into its isolated constituent waveforms to be measured. This process is illustrated for isolating the output response of the fifth pulse in the entire pulse train (shown by the dashed red waveform) as an example. Output waveforms in response to only the first five pulses (original waveform) and first four pulses (subtracted waveform) are captured. The pulse of interest (the fifth pulse) is isolated by subtracting the subtracted waveform from the original waveform which produces the individual contribution of the fifth pulse. The isolated waveform is then saved and categorized based on the order of the pulse (in this case, fifth order). This process is repeated for every individual pulse in the full 20 s input train. **(B)** After all pulses in the input train have been saved and categorized, the average waveform of each order is found. The full duration at half maximum (FDHM) is found for each average *n*th order waveform. The FDHM and average waveform are then used in the optimization of the three time constants for the triple exponential basis waveform of the LUTsyn model as described in Equations **12**, **13**.

From the previously discussed assumption 2, a different set of time constants were used for each different order response. For example, the 4th order LUTsyn model uses four different sets of time constants; one set for each 1st, 2nd, 3rd, and 4th order output response. Optimization of these time constants was performed by first obtaining the average 1st, 2nd, …, *N*th order output responses (using the kinetic models) in a Poisson random pulse train with a mean firing rate of 10 Hz (see Figure 4). The full duration at half maximum (FDHM) was calculated for each of the average *N* output responses to characterize the temporal dynamics of the output waveform of the kinetic models and to optimize the basis waveforms of the LUTsyn model. A grid search algorithm was used to optimize each triplet of time constants for each order. The objective function used in this algorithm was the equally weighted sum of the relative error of the FDHM and the normalized root mean square error (NRMSE) between the response from the kinetic model and estimation of the triple exponential model. The equations below summarize the objective functions used in the time constant optimization:


(12) $$f\,\left({\text{T}}_{c}\right)=\frac{\left|\gamma -\hat{\gamma }\right|}{\gamma }+N\,R\,M\,S\,E$$

(13) $$N\,R\,M\,S\,E=\sqrt{\frac{\sum_{k=0}^{K-1}{\left(y\,\left(k\right)-\hat{y}\,\left(k\right)\right)}^{2}}{\sum_{k=0}^{K-1}y^{2}\,\left(k\right)}}$$

for which *f*(_**Tc**_) represents the objective function that is being minimized, _**Tc**_ is the vector containing the three time constants *T*_*c1*_, *T*_*c2*_, and *T*_*c3*_, γ represents the target FDHM given by the average kinetic response, $\hat{\gamma }$ represents the FDHM given by the triple exponential estimate function using the time constants of _**Tc**_, and *NRMSE* represents the normalized root mean square error between two time series *y*(*k*) (the reference) and $\hat{y}\,\left(k\right)$ (the estimate), both which are of length *K*. In the context of time constant optimization, *y*(*k*) represents the kinetic model’s normalized average output in response to one input event and $\hat{y}\,\left(k\right)$ represents the estimate output response of the normalized triple exponential function, parameterized by _**Tc**_. The time constants optimization was performed *N* times (*N* = 4 for AMPAr and *N* = 5 for NMDAr), according to the objective function above, resulting in *N* triplets of time constants—one triplet for each response order being modeled (see Table 1).

**TABLE 1:** Average full duration at half maximum (FDHM) and optimized time constants.

**Receptor**	**Order**	**Average FDHM (ms)**	***T*_*c1*_ (ms)**	***T*_*c2*_ (ms)**	***T*_*c3*_ (ms)**
AMPAr	1	5.4658	1.05	3.80	16.0
	2	5.6135	1.05	3.95	19.0
	3	5.6232	1.10	3.85	18.0
	4+	5.8301	1.05	4.20	19.5
NMDAr	1	59.7048	18	23	148
	2	59.0823	17	20	140
	3	63.3173	18	21	144
	4	62.2335	18	22	168
	5+	62.6808	18	21	140

*The average FDHMs of the kinetic models over a 20 s 10 Hz input train is reported, which are grouped by pulse order and receptor. The triple exponential time constants are also reported, which optimize the normalized root mean square error (NRMSE) of the waveform and the average FDHM.*

During this study, it was found that the global error between the target and estimated waveforms were not the best predictors of model performance in terms of *spike synchrony*. In many cases, optimizing solely based on NRMSE within the constraint of the triple exponential function resulted in deviations during the decay phase of the output waveform and poor performance in the LUTsyn model’s spike synchrony. Therefore, the FDHM was considered as a way of further constraining and optimizing the temporal aspects of the output waveform and ultimately, spike synchrony.

## Results

### Model Validation—Accuracy

**FIGURE 5:**
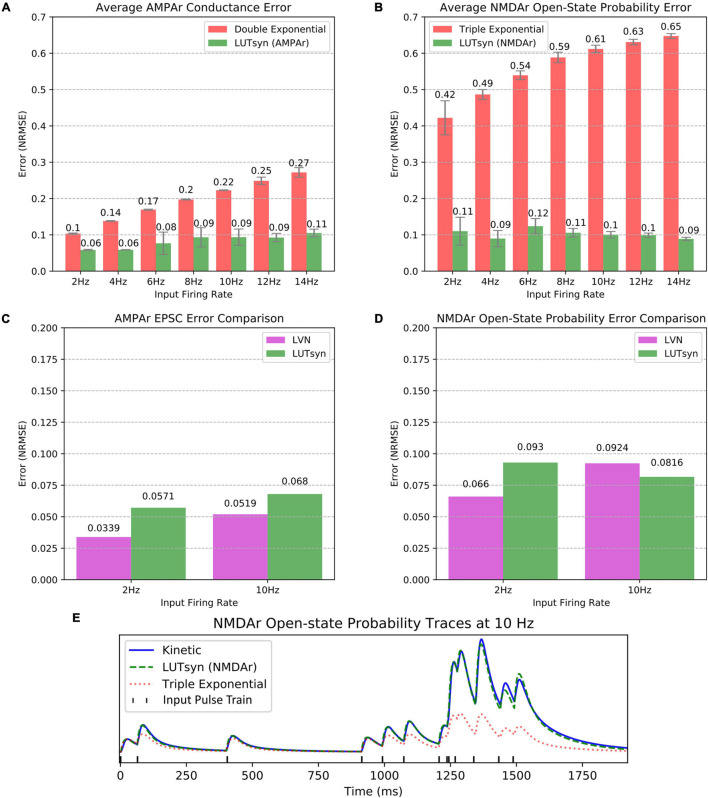
Accuracy comparison of synapse models. **(A,B)** Error of the LUTsyn and exponential models were measured using NRMSE at 2–14 Hz Poisson inputs. The reported NRMSE values were averaged over five different random input trains (each 20 s long). **(A)** Average AMPAr conductance NRMSE is compared. **(B)** Average NMDAr open-state probability NRMSE is compared. **(C,D)** The LUTsyn and LVN models’ accuracy were compared using NRMSE over 2 and 10 Hz (each containing one 20 s trial). The LVN NRMSE values were taken directly from Hu et al. (2018). The LUTsyn values were found by reproducing the same simulations used in Hu et al. (2018). **(C)** AMPAr EPSC NRMSE is compared. **(D)** NMDAr open-state probability NRMSE is compared. **(E)** NMDAr open-state probability sample traces are compared at 10 Hz for the first 2 s of a 20 s simulation.

The LUTsyn model’s accuracy was evaluated using validation datasets consisting of binary Poisson random pulse train inputs with mean firing rates of 2, 4, 6, 8, 10, 12, and 14 Hz, all with simulation durations of 20 s. These pulse trains were chosen to provide a broad spectrum of input patterns that led to a diverse range of non-linearities. The outputs of the validation datasets were the responses given by the AMPAr or NMDAr kinetic models (both coupled with the NTDiffusion mechanism) implemented in NEURON. The outputs that were measured were AMPAr conductance and NMDAr open-state probability. To provide context to our LUTsyn model’s performance, we compared its accuracy to that of our previous LVN model, a double exponential synapse (for AMPAr), and a triple exponential synapse (for NMDAr). Each of the models were given the same validation inputs while accuracy was evaluated with respect to their ability to replicate the outputs of the kinetic models. The metric used for evaluating accuracy was chosen to be the NRMSE. The NRMSE is a good choice of criterion for this data because the baseline of the output is zero. The error calculated using NRMSE is primarily focused on the difference in amplitudes of the expected and predicted response, which is precisely the desired measurement. In this study, we implemented each model into NEURON and simulated a single synapse for each model with fixed timesteps of 0.1 ms. Figures 5A,B show the comparison of average NRMSE results of both AMPAr and NMDAr across mean frequencies ranging from 2 to 14 Hz using the exponential and LUTsyn models. In terms of accuracy, the exponential synapse models performed the worst in every case with NRMSE values ranging from 10 to 27% in AMPAr and 42 to 65% in NMDAr due to their inability to capture any degree of non-linearity while the LUTsyn model maintained NRMSE values ranging from 6 to 11% in AMPAr and 9 to 12% in NMDAr. Figures 5C,D show an accuracy comparison of the LUTsyn and LVN models. The LUTsyn model provided marginally worse results than the LVN model except for the 10 Hz NMDAr case. Despite small decreases in accuracy compared to the LVN model, the LUTsyn model still provides accurate results that are adequate in replicating kinetic model dynamics. Figure 5E illustrates a sample comparison of the NMDAr outputs of open-state probability simulated at 10 Hz using the kinetic, LUTsyn, and triple exponential synapse models. These output traces make it clear that the non-linear dynamics of NMDAr cannot be accurately captured by the linear triple exponential synapse model but can be captured by the LUTsyn model.

### Model Validation—Spike Synchrony

**FIGURE 6:**
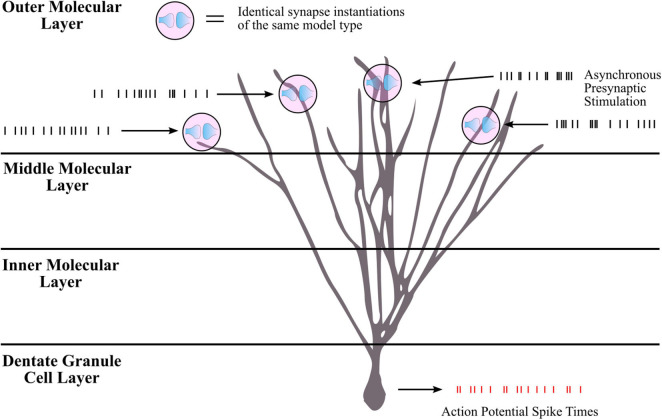
Simulation protocol for measuring spike times. Spike synchrony was assessed by first collecting spike times for all synapse model-firing rate combinations. The simulation protocol was implemented in NEURON with a single rat dentate granule cell consisting of thousands of synapses distributed in the outer third molecular layer. For a given simulation, all synapse instantiations were of the same model type (all kinetic, all LUTsyn, etc.) and each synapse was stimulated with the same mean firing rate. Input firing rates ranged from 2 to 14 Hz. For a given input firing rate, each synapse received a unique Poisson process stimulation pattern with the same mean frequency, i.e., the inputs were asynchronous across synapses. The stimulation pattern that a synapse received remained the same regardless of the synaptic model being used such that if the synapse models performed identically, the granule cell should elicit the same spiking train. Membrane voltage at the soma was recorded as well as times that action potentials were produced (i.e., “spike times”). Spike times were collected for all synapse models across all input firing rates. The spike times produced by the kinetic models were used as reference in assessing the other models’ spike synchrony (see Figure 7 for comparison results).

**FIGURE 7:**
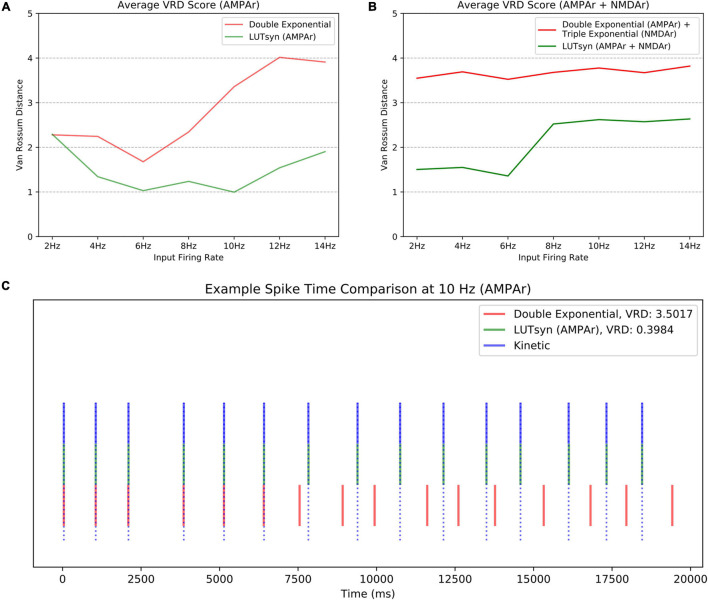
Comparison of spike synchrony. Spike synchrony was assessed and compared for each synaptic model over multiple 20 s simulations over multiple input firing rates. The van Rossum Distance (VRD) was used as the metric for quantifying spike synchrony, which uses the kinetic models’ spike times as reference. A low VRD score reflects pairs of spike trains that are temporally similar (i.e., high spike synchrony). The VRD scores shown above reflect average scores over five unique trials (i.e., five different random seeds). **(A)** The average VRD for simulations only containing AMPAr models are compared. **(B)** The average VRD for simulations containing both AMPAr and NMDAr models are compared. **(C)** An example trial of spike times, simulated at 10 Hz with only AMPAr synapses, is shown as an aid for spike time-VRD score visualization.

Though the LUTsyn models had been shown to perform better than the exponential models at the receptor and conductance level, the errors were non-zero. One of the ultimate goals of this work is to evaluate how the lower-level molecular differences are propagated through a neural system and consequently affect higher-level properties, such as spike timing at the cellular level. Therefore, we utilized *spike synchrony* to quantify the differences in spike timing that would result from using the various synaptic models and to evaluate the LUTsyn model’s capability of replicating the behavior of the kinetic models. A NEURON simulation protocol was devised to measure spike synchrony which included a model of a single granule cell from the rat dentate gyrus with a varying number of active input synapses ranging from 1,200 to 6,000 (depending on mean input firing rate) at the outer molecular layer (see Figure 6). The granule cell morphology was reduced to an equivalent four compartment model, as described in Yu et al. (2019), using the reduction algorithm outlined in Marasco et al. (2012). The number of input synapses were changed based on input firing rate so that regardless of the input firing rate, the granule cell output firing rate was approximately the same (see Table 2). Fixed timesteps of 0.1 ms were used. Within a given simulation, all the synapses were given unique 20 s Poisson random input trains of the same mean firing rate of either: 2, 4, 6, 8, 10, 12, or 14 Hz. A spike detection algorithm was used on the granule cell which recorded the times the soma produced an action potential (i.e., the “spike times”), which was defined as the time at which the somatic voltage crossed 0 mV during a rising phase. Spike times were generated and compared using the different synapse models and the spike times generated by the kinetic models were used as reference. The spike synchrony was evaluated in two cases: (1) synapses containing only AMPAr and (2) synapses containing both AMPAr and NMDAr. In simulations including synapses that contained both AMPAr and NMDAr, analogous pairs of models were chosen to be simulated together (e.g., LUTsyn model for AMPAr was simulated with LUTsyn model for NMDAr). An efficient version of the van Rossum distance (VRD) (Van Rossum, 2001; Houghton and Kreuz, 2012) was used to measure spike synchrony. The input trains for each synapse were randomly generated over five different seeds such that five different VRD measurements were taken for each input firing rate. The five VRD scores were then averaged (per firing rate) and compared among the different synapse models. Figure 7 compares the average VRD score of the exponential and LUTsyn synapse models as a function of input firing rate. The results demonstrate that in all cases, the LUTsyn model provided superior spike synchrony and replicated kinetic spike times better than the exponential models—except in the 2 Hz AMPAr case, in which both models gave nearly identical VRD scores. This is likely due to the fact that AMPAr dynamics occur fast in comparison with a 2 Hz firing rate, thus, resulting in low spike orders and the LUTsyn synapses behaving quite linearly. In the case of cells containing both LUTsyn AMPAr and NMDAr, spike synchrony noticeably degrades as input firing rate increases (Figure 7B). This is likely due to the non-linearities that arise due to pulse interactions that are not accounted for by the NMDAr LUTsyn model. NMDAr dynamics take significantly longer and require a longer memory window than AMPAr. This longer 1,000 ms memory window, combined with the increased firing rates, inherently increase the likelihood that pulses beyond the LUTsyn’s limit of 5th order are not accounted for resulting in uncaptured non-linear pulse interactions, and thus, higher error.

**TABLE 2:** Number of synapses simulated for each input firing rate in NEURON for measuring spike synchrony.

**Input firing rate (Hz)**	**Number of synapses**
2	6,000
4	3,000
6	2,000
8	1,500
10	1,400
12	1,300
14	1,400

### Performance in Large-Scale Simulations

**FIGURE 8:**
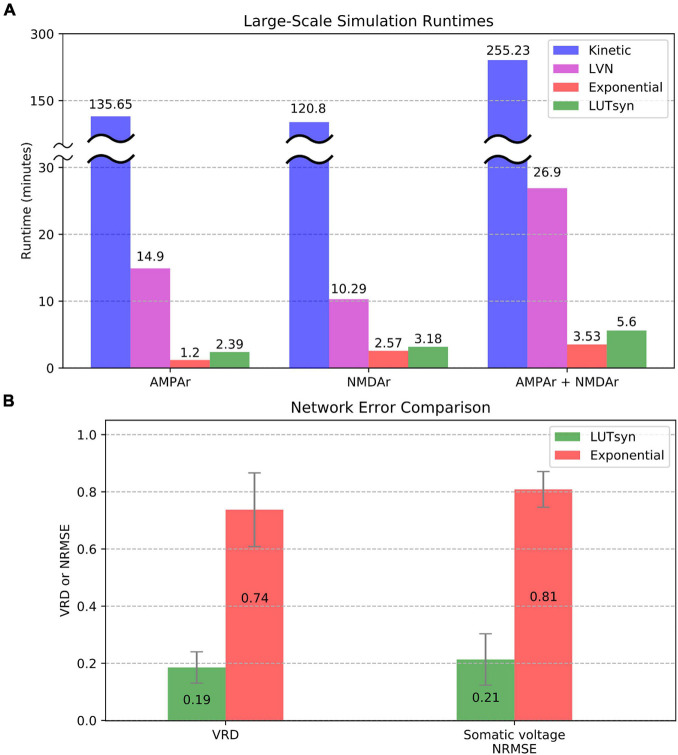
Comparison of large-scale simulations. **(A)** Runtimes were averaged and compared over 10 trials. AMPAr (left) and NMDAr (center) models were first ran individually from each other. Then, both receptors were ran together (right). The kinetic models took about 2 h (receptors simulated individually) to 4 h (receptors simulated together) while the LVN, exponential, and LUTsyn models took minutes. The simulations containing both AMPAr and NMDAr have runtimes that are roughly the sum of their individual constituents. **(B)** Network-level accuracy was compared over 1,200 granule cells by measuring spike synchrony (VRD) and somatic voltage (NRMSE) in reference to kinetic synapses. AMPAr and NMDAr were simulated simultaneously with a 10 Hz input firing rate.

To evaluate the efficiency of each method in large-scale neuronal networks, the runtime was measured when each synapse model was placed into a large-scale setting. The large-scale simulations consisted of entorhinal cortex projections to the dentate gyrus of a rat hippocampus, as described and developed in Yu et al. (2018), with 500 ms of simulated time and fixed timesteps of 0.1 ms. The simulations contained approximately a thousandth of the number of connections in a single hemisphere of a rat’s entorhinal-dentate system, which contained 46,000 cells from the lateral entorhinal cortex, 66,000 cells from the medial entorhinal cortex, and 1,200 granule cells of the dentate gyrus resulting in a total of 3,417,600 synapses. Again, the granule cells were reduced to models containing four compartments as described in the previous section and carried out in Yu et al. (2019). The University of Southern California’s Center for Advanced Research Computing (CARC) was used for carrying out the large-scale simulations. Parallelization of the simulation was performed using 48 processing cores, each with 3 GB of allocated RAM. Each CPU was an Intel Xeon 4116 Processor with a clock frequency of 2.10 GHz. Figure 8A compares the large-scale simulation runtimes when every synapse instantiation is changed to either the kinetic, LVN, LUTsyn, or exponential synapse models. Runtimes were measured in three cases: only AMPAr synapses, only NMDAr synapses, and synapses containing both AMPAr and NMDAr. The results show the extreme slowdown of the kinetic models, which took over 2 h to simulate AMPAr and NMDAr individually and over 4 h to simulate them together. The LVN synapse model had a considerable speed-up with runtimes ranging from ∼10 to ∼27 min. The LUTsyn method provided a vast speed advantage over the kinetic and LVN models with runtimes ranging from ∼2.4 to ∼5.6 min. Furthermore, these speeds were similar to those of the exponential models with runtimes that were around a minute longer in the individual receptor cases. Accuracy at the level of the large-scale network was also evaluated (Figure 8B). Spike synchrony (VRD) and somatic voltage (NRMSE) were compared (averaged over 1,200 granule cells) between the exponential models and LUTsyn models in reference to the kinetic models (when both AMPAr and NMDAr were simulated simultaneously) with input firing rates of 10 Hz. The LUTsyn models demonstrated significantly superior results in both metrics over the exponential models: average VRD of 0.19 vs. 0.74 and average NRMSE of 0.21 vs. 0.81. It should be noted that the values of VRD obtained in this section were considerably smaller than those found in Figure 7 due to the simulations being much shorter (500 ms vs. 20 s). The longer simulated time in the previous section allowed for more desynchronization of spike times, allowing errors to propagate and become more apparent as time progressed, and thus, produced a larger VRD value. These results in runtime efficiency and network-level accuracy indicate that the LUTsyn model maintains the predictive power of the kinetic synapse models while greatly reducing their complexity and computational load.

## Discussion

Multi-scale and large-scale models of the nervous system have great potential in revealing insights on how micro-level interactions may lead to emergent effects observed at the neuronal network level, and how network level dynamics affect subcellular molecular pathways. Utilization of such models would have powerful implications in healthcare as they would enable *in silico* investigations of neurological disorders and facilitate identification and development of novel therapeutics, offering an inexpensive and efficient complement to animal models. Currently, the limits of large-scale modeling over multiple hierarchies are broadly constrained by the computational power of today’s technology and the computational load that is imposed by the model itself. In this report, we addressed the latter by circumventing model complexity with simple memory access.

From a scientific perspective, mechanistic approaches are excellent investigative tools that provide highly accurate representations of biological systems, which lead to an improved understanding of them. As discussed, kinetic state models are utilized to depict synaptic dynamics with high fidelity. However, from an engineering perspective, such approaches become increasingly impractical in large-scale settings as the number of synapses becomes large and due to the large number of differential equations used in kinetic models, which lead to significant computational complexity. In this study, we proposed an efficient look-up table approach that replicates kinetic model properties while reducing computational load, thereby bridging hierarchical gaps in multi-scale and large-scale modeling.

We have shown that the LUTsyn model can successfully reproduce the kinetic model properties in a highly parsimonious fashion. At the molecular/conductance level, we compared our LUTsyn model’s accuracy against the accuracy of our previous LVN and linear exponential synapse models over 2 and 10 Hz random pulse trains. We have found that the LUTsyn models performed with similar accuracy as the LVN models, and in one instance, had a slight improvement over them. As expected, the exponential synapse models performed poorly in replicating kinetic level non-linearity, resulting in high error. Additionally, we measured and compared the average spike synchrony of the synapse models which demonstrated that higher/cellular level spike timings were preserved using the LUTsyn model. Finally, we assessed how well the LUTsyn model facilitates simulations in large-scale neuronal networks by measuring its runtime and comparing it to other synapse models. The results showed that the kinetic models were remarkably slower than the other methodologies. Although the LVN models had a large speed advantage over the kinetic models, the LUTsyn and exponential models yielded the fastest runtimes overall. Despite the marginal decrease in accuracy when compared to the predecessor LVN model, the gain in speed makes the LUTsyn model a powerful middle-ground alternative in enabling large-scale simulations.

Our LUTsyn model is an input-output approach that aims to circumvent computationally slow calculations with comparably fast memory access. The simplicity of the look-up table approach makes it a highly generalizable method that can be utilized across other non-linear time-variant systems; its advantages are maximized in applications that require many identical model instantiations to be simulated. Any biological system that shares a common waveform as its output with varying amplitudes depending on the precise timings of past stimuli could be simplified to a look-up table model to alleviate computational load. As an input-output model, the look-up table approach is only concerned with the outcomes of the system and not its working details, making it invariant to complexity. Despite the LUTsyn model’s ability to drastically reduce model complexity, it is important to note its current limitations. First, multi-scale models that incorporate synapses using the LUTsyn model will not capture any biophysical changes that result from signal cascades outside of the receptor (i.e., LUTsyn instantiations will have a fixed behavior). This limits the ability to investigate feedback mechanisms that may affect synaptic strength (e.g., long-term potentiation and depression) and their subsequent impacts on higher levels in the hierarchical chain. Another shortcoming of the LUTsyn model is that a new look-up table must be generated and loaded into memory for every different type (or configuration) of synapse that must be simulated. Two obvious examples would be in the modeling of neurological perturbations and/or drug interactions which would result in changes in synaptic behavior. These would consequently lead to the elaboration of additional look-up tables. For simulations involving many types of synapses, computer memory may become an issue due to the amount of data that must be loaded for all the different look-up tables. Furthermore, the assumptions that underlie the LUTsyn model may lead to inaccuracies in higher firing rate simulations. For example, the order (*N*) of the model determines the maximum number of past pulses that will be considered when predicting the present output response. It is possible in higher firing rate situations that the non-linear effects emerging from pulses, which are not accounted for, are not captured. One way to address this problem would be to increase the model’s order; however, doing so would greatly increase the size of the look-up table by an amount that is similar to increasing the dimension of a multi-dimensional array. Moreover, the LUTsyn model imposes the simplifying assumption that the normalized output waveform for each response order has an identical shape. In general, this is not the case, which may lead to further errors propagating in situations that contain a large variety of non-linear interactions and variations in waveform dynamics.

Regardless of the limitations described above, its underlying approach successfully highlights the advantages taken from both mechanistic (biological realism) and input-output modeling (fast runtimes). It has demonstrated the ability to preserve synaptic dynamics while drastically reducing complexity by implicitly abstracting non-linearity in the form of look-up tables. We have demonstrated its powerful capabilities in replicating complex mechanistic synapse dynamics at remarkable speeds with a low computational cost. The LUTsyn model’s ability to reproduce biologically realistic synapses while maintaining high parsimony makes it a strong candidate in large-scale modeling of neuronal networks that span multiple hierarchies.

## Copyright Material

The software described in this manuscript is copyrighted by the University of Southern California. Permission will be granted to use, copy, modify, and distribute this software and its documentation for educational, research, and non-profit purposes, without fee under specific conditions; you may contact the authors for more information. Permission to make commercial use of this software may be obtained by contacting: *USC Stevens Center for Innovation*, *University of Southern California*, *1150 S. Olive Street, Suite 2300*, *Los Angeles, CA 90115, United States*. The full copyright is provided with the software and upon request.

## Data Availability Statement

The source code of the models, along with example scripts, is available at: http://modeldb.yale.edu/267103.

## Author Contributions

D-TP developed and implemented the synapse models, performed the analysis, and produced the manuscript. GY provided the network model framework for large-scale simulations, provided guidance, and assisted in preparing the manuscript. J-MB provided the initial idea of the project, provided guidance, and assisted in preparing the manuscript. TB oversaw the project and worked to maintain funding to support the project.

## Conflict of Interest

The authors declare that the research was conducted in the absence of any commercial or financial relationships that could be construed as a potential conflict of interest.

## Publisher’s Note

All claims expressed in this article are solely those of the authors and do not necessarily represent those of their affiliated organizations, or those of the publisher, the editors and the reviewers. Any product that may be evaluated in this article, or claim that may be made by its manufacturer, is not guaranteed or endorsed by the publisher.

## Supplementary Material

Supplementary Figure 1 Schematic of AMPAr kinetic state model adapted from Robert and Howe, (2003).

Supplementary Figure 2 Schematic of NMDAr kinetic state model adapted from Schorge et al., (2005).

Supplementary Figure 3 Effects of LUT granularity. **(A)** LUTsyn AMPAr conductance error as a function of input firing rate is compared across multiple look-up tables with varying granularities. NRMSE values are averaged over five trials, each lasting 20 s long. Accuracy improvement is negligible when changing from 1 to 0.5 ms granularities. **(B)** The amount of memory consumed from a fourth-order look-up table (*M* = 300 ms) as a function of its granularity is shown. The y-axis is shown in log scale. Although the accuracy change is negligible between 1 and 0.5 ms granularities, the amount of memory consumed is about an eightfold increase.

Supplementary Table 1 Rate constants of AMPAr kinetic state model.

Supplementary Table 2 Rate constants of NMDAr kinetic state model.
