# Supplementary figures and images for: Bridging Hierarchies in Multi-Scale Models of Neural Systems: Look-Up Tables Enable Computationally Efficient Simulations of Non-linear Synaptic Dynamics
Source: Front Comput Neurosci. 2021 Oct 1;15:733155. doi: 10.3389/fncom.2021.733155 (PMC8517488; doi:10.3389/fncom.2021.733155)

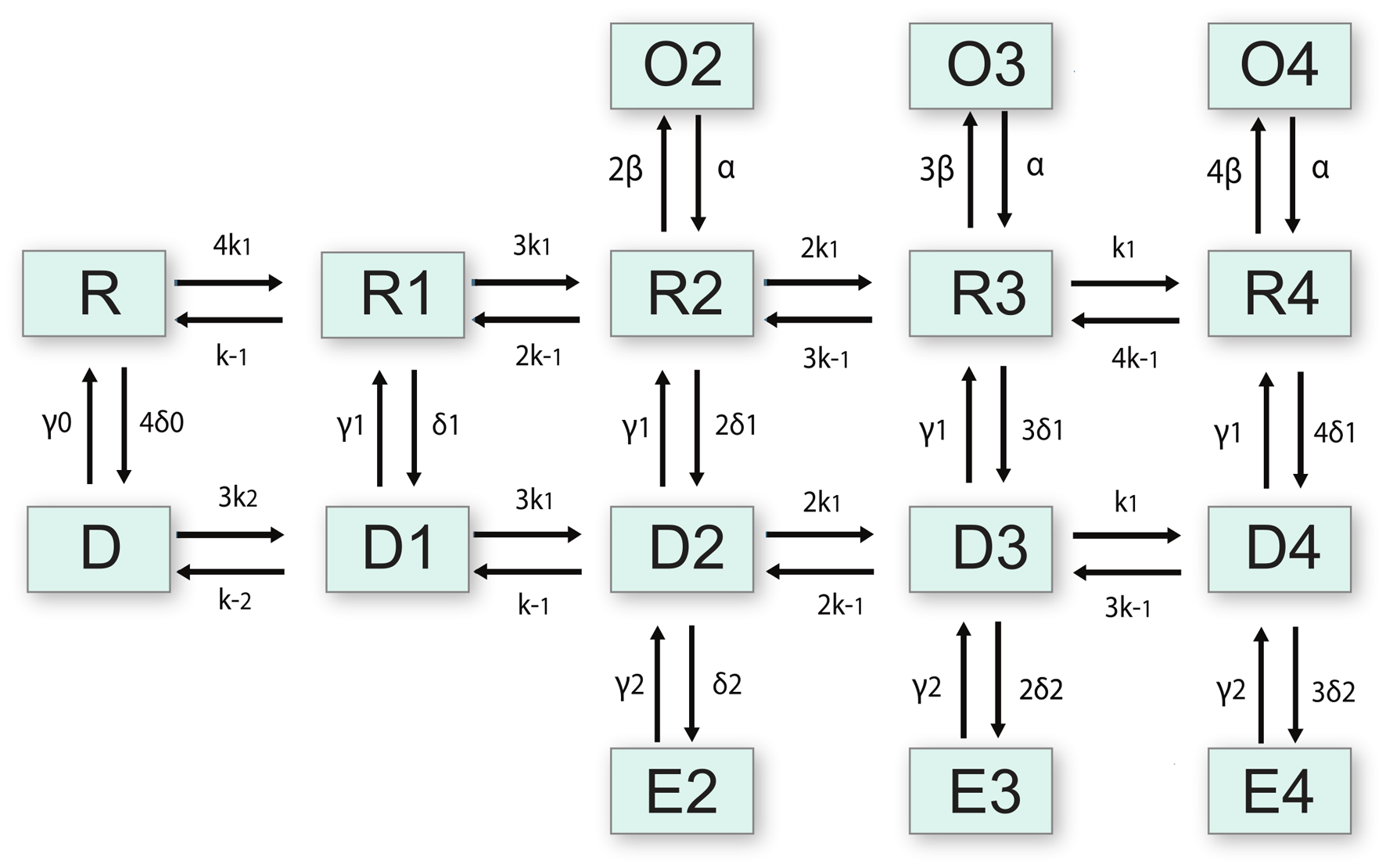

Supplement: Supplementary Figure 1 — Schematic of AMPAr kinetic state model adapted from Robert and Howe, (2003). [file Image_1.png]

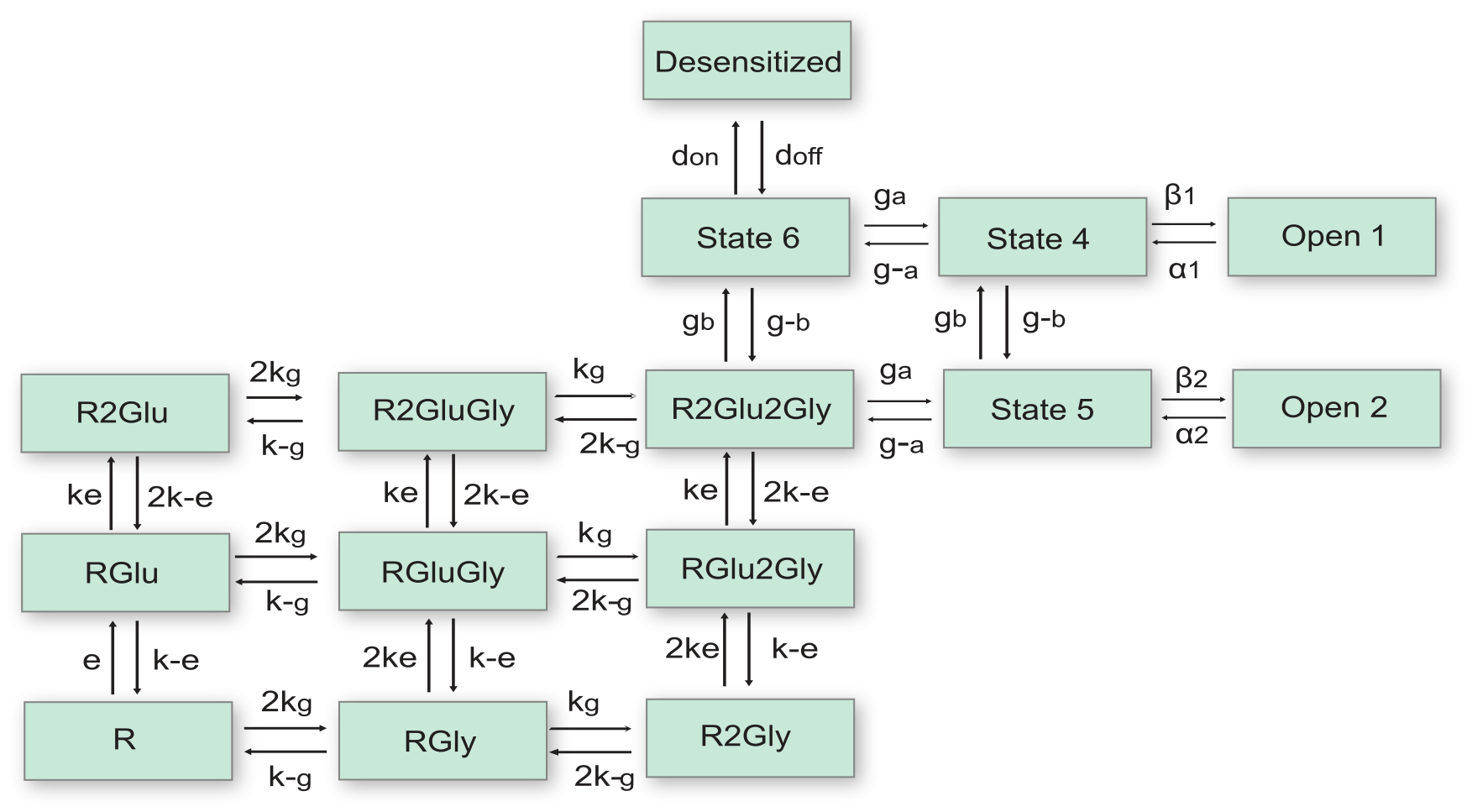

Supplement: Supplementary Figure 2 — Schematic of NMDAr kinetic state model adapted from Schorge et al., (2005). [file Image_2.png]

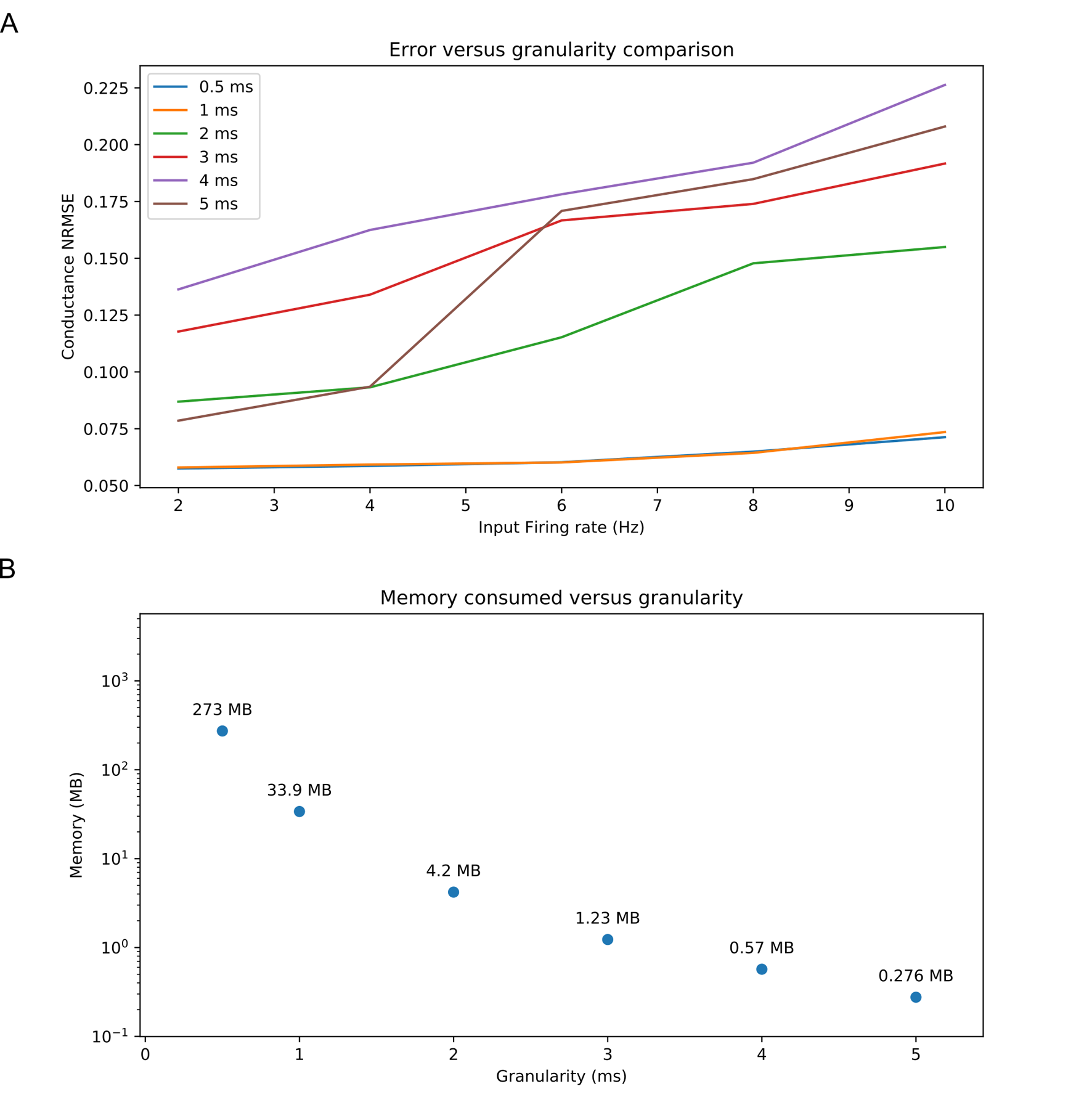

Supplement: Supplementary Figure 3 — Effects of LUT granularity. (A) LUTsyn AMPAr conductance error as a function of input firing rate is compared across multiple look-up tables with varying granularities. NRMSE values are averaged over five trials, each lasting 20 s long. Accuracy improvement is negligible when changing from 1 to 0.5 ms granularities. (B) The amount of memory consumed from a fourth-order look-up table (M = 300 ms) as a function of its granularity is shown. The y-axis is shown in log scale. Although the accuracy change is negligible between 1 and 0.5 ms granularities, the amount of memory consumed is about an eightfold increase. [file Image_3.png]
