# Supplementary material for: Bridging Hierarchies in Multi-Scale Models of Neural Systems: Look-Up Tables Enable Computationally Efficient Simulations of Non-linear Synaptic Dynamics
Source: Front Comput Neurosci. 2021 Oct 1;15:733155. doi: 10.3389/fncom.2021.733155 (PMC8517488; doi:10.3389/fncom.2021.733155)
Supplement: Supplementary Table 1 — Rate constants of AMPAr kinetic state model. [file Table_1.docx]

**Table A1 | Rate constants of AMPAr kinetic state model**

| Kinetic AMPAr rate constants | |
| --- | --- |
| Rate constant | Value |
| *k_1_* | 10 mM^-1^ ms^-1^ |
| *k_-1_* | 7 ms^-1^ |
| *k_2_* | 10 mM^-1^ ms^-1^ |
| *k_-2_* | 0.00041 ms^-1^ |
| *γ_0_* | 0.001 ms^-1^ |
| *δ_0_* | 3.3e-06 ms^-1^ |
| *γ_1_* | 0.42 ms^-1^ |
| *δ_1_* | 0.017 ms^-1^ |
| *γ_2_* | 0.2 ms^-1^ |
| *δ_2_* | 0.035 ms^-1^ |
| *β* | 0.55 ms^-1^ |
| *α* | 0.3 ms^-1^ |
