# Supplementary material for: Bridging Hierarchies in Multi-Scale Models of Neural Systems: Look-Up Tables Enable Computationally Efficient Simulations of Non-linear Synaptic Dynamics
Source: Front Comput Neurosci. 2021 Oct 1;15:733155. doi: 10.3389/fncom.2021.733155 (PMC8517488; doi:10.3389/fncom.2021.733155)
Supplement: Supplementary Table 2 — Rate constants of NMDAr kinetic state model. [file Table_2.docx]

**Table A2 | Rate constants of NMDAr kinetic state model**

| Kinetic NMDAr rate constants | |
| --- | --- |
| Rate constant | Value |
| *k_e_* | 1.0 mM^-1^ ms^-1^ |
| *k_-e_* | 0.0263 ms^-1^ |
| *k_g_* | 10.0 mM^-1^ ms^-1^ |
| *k_-g_* | 0.0291 ms^-1^ |
| *g_b_* | 0.1681 ms^-1^ |
| *g_-b_* | 0.2632 ms^-1^ |
| *g_a_* | 0.1 ms^-1^ |
| *g_-a_* | 217.6 ms^-1^ |
| *β_1_* | 3.5 ms^-1^ |
| *α_1_* | 0.1744 ms^-1^ |
| *β_2_* | 7.119 ms^-1^ |
| *α_2_* | 4.367 ms^-1^ |
| *d_on_* | 0.0422 ms^-1^ |
| *d_off_* | 0.0129 ms^-1^ |
